# Platelet desialylation correlates with efficacy of first-line therapies for immune thrombocytopenia

**DOI:** 10.1186/s13045-017-0413-3

**Published:** 2017-02-08

**Authors:** Lili Tao, Qingshu Zeng, June Li, Miao Xu, Jiajia Wang, Ying Pan, Huiping Wang, Qianshan Tao, Yang Chen, Jun Peng, Ming Hou, Arend Jan Gerard Jansen, Heyu Ni, Zhimin Zhai

**Affiliations:** 1 Department of Hematology, The Second Affiliated Hospital of Anhui Medical University, Hefei, 230601 China; 2 The Hematological Research Center of Anhui Medical University, Hefei, 230601 China; 3 Department of Hematology, The First Affiliated Hospital of Anhui Medical University, Hefei, 230022 China; 4 Toronto Platelet Immunobiology Group, Keenan Research Centre for Biomedical Science and Department of Laboratory Medicine of St. Michael’s Hospital, Toronto, Canada; 5 Canadian Blood Services, Ottawa, Canada; 6 Department of Laboratory Medicine and Pathobiology, University of Toronto, Toronto, Canada; 7 Department of Hematology and Shandong Provincial Key Laboratory of Immunohematology, Qilu Hospital, Shandong University, Jinan, China; 8 Department of Hematology, Erasmus MC, Rotterdam, The Netherlands; 9 Department of Plasmaproteins Sanquin-AMC Landsteiner Laboratory, Amsterdam, The Netherlands; 10 Department of Physiology, and Department of Medicine, University of Toronto, Toronto, Canada

**Keywords:** Platelet, Immune thrombocytopenia, Antibody, Desialylation, Steroid and IVIG therapy

## Abstract

Immune thrombocytopenia (ITP) is a common autoimmune bleeding disorder. Despite considerable investigation, the pathogenesis of ITP remains incompletely understood, and for many patients, effective therapy is still unavailable. Using murine models and in vitro studies of human blood samples, we recently identified a novel Fc-independent platelet clearance pathway, whereby antibody-mediated desialylated platelets can be cleared in the liver via asialoglycoprotein receptors, leading to decreased response to standard first-line therapies targeting Fc-dependent platelet clearance. Here, we evaluated the significance of this finding in 61 ITP patients through correlation of levels of platelet desialylation with the efficacy of first-line therapies. We found that desialylation levels between different responses to treatment groups were statistically significant (*p* < 0.01). Importantly, correlation analysis indicated response to treatment and platelet desialylation were related (*p* < 0.01), whereby non-responders had significantly higher levels of platelet desialylation. Interestingly, we also found secondary ITP and certain non-ITP thrombocytopenias also exhibited significant platelet desialylation compared to healthy controls. These findings designate platelet desialylation as an important biomarker in determining response to standard treatment for ITP. Furthermore, we show for the first time platelet desialylation in other non-ITP thrombocytopenias, which may have important clinical implications and deserve further investigation.

**Electronic supplementary material:**

The online version of this article (doi:10.1186/s13045-017-0413-3) contains supplementary material, which is available to authorized users.

## Introduction

Immune thrombocytopenia (ITP) is a common clinical bleeding disorder characterized by an immune-mediated clearance of autologous platelets, predominantly through autoantibodies targeting platelet surface receptors GPIIbIIIa and/or GPIb-IX and clearance by phagocytic cells in the reticuloendothelial system via Fcγ-receptors [1–4]. Low platelet counts place ITP patients at risk for severe bleeding including fatal intracranial hemorrhage. Most therapies for ITP including first-line corticosteroids and immunoglobulin G (IVIG), and last resort splenectomy, mainly target the Fc-dependent clearance pathway via blocking/attenuating Fc-Fcγ-R interaction or removal of putative site of platelet clearance [4]. However, the pathogenesis and mechanisms of therapies remain poorly understood and around 15–20% of ITP patients are inexplicably refractory to first-line therapies, and around 10% are refractory to splenectomy [5, 6]. In recent years, murine models and large cohort human studies report antibody specificity (i.e., anti-GPIIbIIIa versus anti-GPIb-IX) may play a significant role in dictating response to therapy in ITP [7–9]; whereby presence of anti-GPIb-IX antibodies results in decreased response to corticosteroids and IVIG [7–9]. Most recently, we reported that anti-GPIbα and some anti-GPIIbIIIa antibodies in humans induced platelet desialylation leading to Fc-independent platelet clearance in the liver via hepatic asialoglycoprotein Ashwell-Morell receptors [10], suggesting antibody-mediated desialylation may be one of the underlying mechanisms behind resistance to standard ITP therapies [8, 9, 11].

**Fig. 1:**
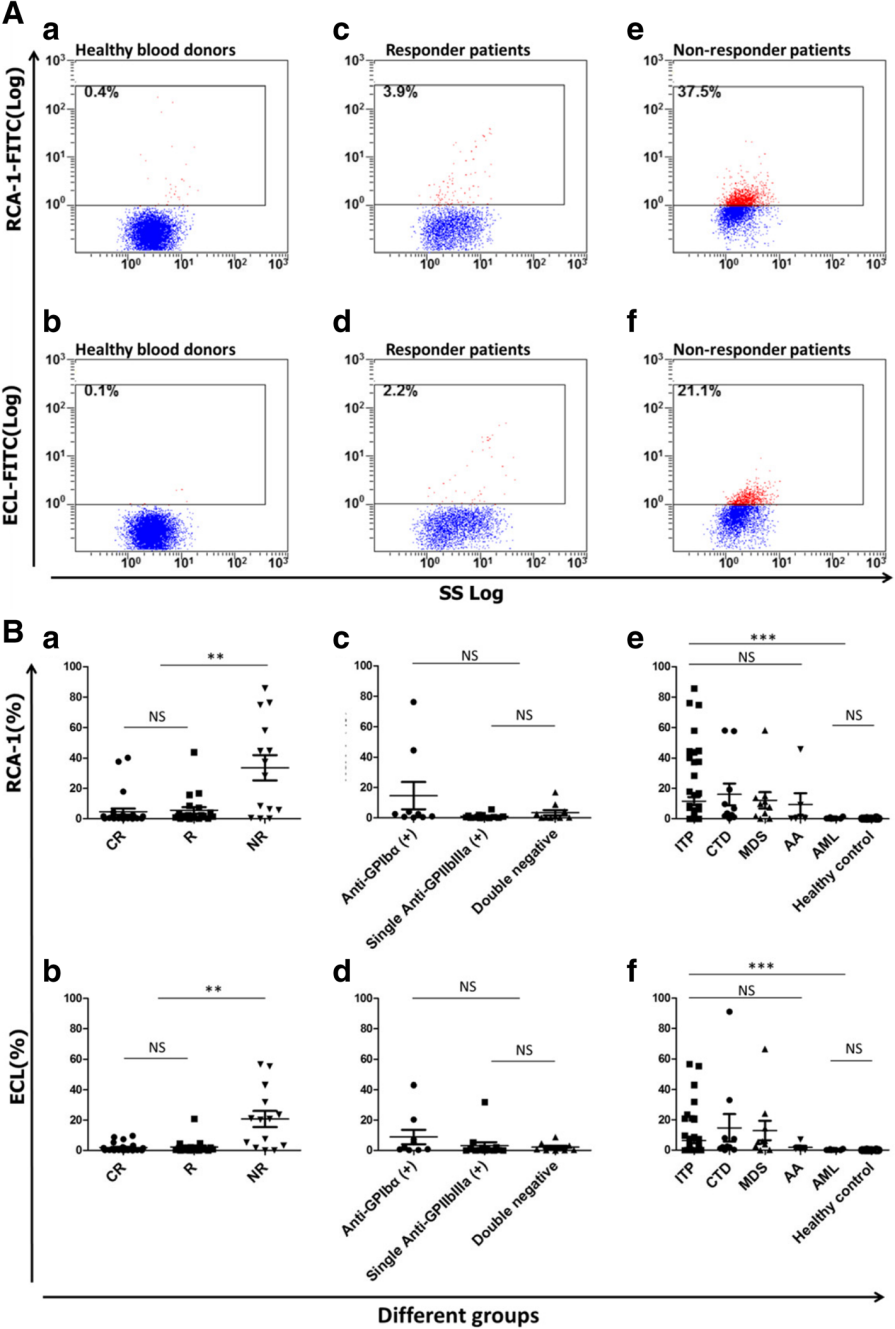
**A** Platelet desialylation in representative patients and healthy controls. The RCA-1 and ECL binding to platelets from healthy blood donors (**a**, **b**), responder (**c**, **d**), and non-responder patients (**e**, **f**) were examined by flow cytometry. The representative dot plots from each group are shown. **B** Platelet desialylation in different patient groups and healthy controls. The RCA-1 and ECL levels (Mean ± SEM) in complete responders (CR), responders (R) and non-responders (NR) (**a**, **b**); anti-GPIbα antibody positive (+) group, single anti-GPIIb/IIIa antibody (+) group, and double negative group (**c**, **d**); and in the thrombocytopenias/controls (**e**, **f**) (ITP, CTD, MDS, AA, AML, and healthy controls) were examined by flow cytometry. Each point represents the level of platelet desialylation of an individual patient or healthy blood donor (**p < 0.01; ***p < 0.001)

In the present study, we sought to address whether increased platelet desialylation was correlated with decreased response to treatment in ITP patients (Additional file 1: Supplementary Material). The platelets of randomly and consecutively enrolled 61 patients diagnosed with primary ITP were tested for desialylation prior to the indicated treatments (Table 1). Fluorescein-conjugated lectins *Ricinus communis* agglutinin I (RCA-1) and *Erythrina cristagalli* lectin (ECL) were used to detect desialylated galactose and β-GlcNAc residues via flow cytometry. We found the platelets of ITP patients had significantly higher desialylation as measured by both RCA-1 and ECL binding compared to those of healthy blood donors (*p* < 0.05) (Fig. 1). The 61 ITP patients subsequently underwent standard first-line therapy independent of platelet desialylation and MAIPA assays. After 1 month of treatment, there were 26 complete responders (CR), 21 responders (R), and 14 non-responders (NR) (Table 1). Retrospective data analysis using Kruskal-Wallis rank sum test revealed NR patients had significantly higher platelet desialylation, as compared to the CR and R groups (*p* < 0.01). Correlation analysis indicated that efficacy and the desialylation level are related (RCA-1 *r* = 0.395, *p* < 0.01; ECL *r* = 0.391, *p* < 0.01). The higher desialylation, the poorer the efficacy of therapy observed.

**Table 1:** Platelet desialylation of different groups [*M* (*P*_25_, *P*_75_)]

	Age	Gender (M/F)	PLT (×10^9^/L)	RCA-1 (%)	ECL (%)
ITP (*n* = 61)	43 ± 18	18/43	16.0 ± 12.5	1.60 (0.50,8.50)	1.30 (0.30,5.05)
Efficacy grouping (*n* = 61)					
CR (*n* = 26)	36 ± 16	4/22	16.1 ± 15.3	1.10 (0.30,2.05)	0.85 (0.28,1.90)
R (*n* = 21)	44 ± 19	10/11	17.2 ± 10.5	1.80 (0.65,5.75)	1.00 (0.30,2.05)
NR (*n* = 14)	52 ± 17	4/10	13.9 ± 9.7	32.95 (4.40,62.20)	20.60 (2.83,34.68)
Antibody grouping (*n* = 33)					
Anti-GPIbα (+) (*n* = 9)	39 ± 14	2/7	10.7 ± 5.3	2.50 (0.55,24.15)	2.20 (0.45,13.85)
Single anti-GPIIbIIIa (+) (*n* = 14)	35 ± 16	3/11	16.0 ± 14.5	0.55 (0.18,1.70)	0.35 (0.10,1.90)
Double negative (*n* = 10)	41 ± 17	5/5	14.9 ± 9.7	0.65 (0.10,5.50)	1.15 (0.10,2.15)
CTD (*n* = 10)	43 ± 20	3/7	20.3 ± 20.0	5.15 (1.63,28.85)	2.20 (0.90,14.25)
MDS (*n* = 10)	51 ± 27	3/7	29.3 ± 18.4	8.75 (1.30,14.03)	5.60 (2.08,16.85)
AA (*n* = 6)	31 ± 11	4/2	28.2 ± 9.6	0.75 (0.18,18.3)	0.95 (0.10,3.05)
AML (*n* = 8)	49 ± 19	4/4	19.4 ± 18.6	0.20 (0.13,0.80)	0.03 (0.01,0.50)
Healthy control (*n* = 20)	41 ± 12	10/10	197.7 ± 61.7	0.10 (0.10,0.30)	0.00 (0.00,0.10)

The platelets of primary ITP patients were collected prior to treatment. Fluorescin-conjugated lectins RCA-1 and ECL were used to detect desialylated galactose and β-GlcNAc residues on platelets via flow cytometry. Platelets from healthy blood donors (controls) and secondary ITP and non-ITP thrombocytopenic patients were also studied. Normal distribution measurement data is presented as mean ± SEM; skewed distribution measurement data is presented as *M* (*P*_25_, *P*_75_), in which *M* represents the median, *P*_25_ and *P*_75_ represent the 25th percentile and 75th percentile, respectively. Kruskal-Wallis rank sum test showed platelet desialylation is significantly higher in ITP patients as compared to that in healthy blood donors (RCA-1 *Z* = −4.918, *p* < 0.001; ECL *Z* = −5.512, *p* < 0.001). The course of therapies was independent from the platelet desialylation assays. The RCA-1 and ECL-positive platelets in non-responder (NR) group are significantly higher than those in complete responder (CR) and responder (R) groups (RCA-1 χ^2^ = 10.581, *p* < 0.01; ECL χ^2^ = 13.741, *p* < 0.005). No significant difference was observed between CR and R groups (*p* > 0.05). Correlation analysis indicated that as platelet desialylation increases, the efficacy of therapy decreases. Higher platelet desialylation in ITP patients with anti-GPIbα antibodies was observed as compared with other ITP patients although statistical difference was not reached (RCA-1 χ^2^ = 3.729, 0.16 > *p* > 0.05; ECL χ^2^ = 3.864, 0.15 > *p* > 0.05). Higher levels of platelet desialylation were also observed in patients of CTD (systemic lupus erythematosus, *n* = 6; sicca syndrome, *n* = 4) with thrombocytopenia; MDS and AA as compared with healthy controls (RCA-1 χ^2^ = 33.790, *p* < 0.001; ECL χ^2^ = 42.992, *p* < 0.001). There is no statistical difference in platelet desialylation between the AML patients and healthy donors (*p* > 0.05)

To test whether the presence of anti-GPIbα antibodies is associated with the platelet desialylation, we detected antibody using MAIPA in the available 33 patient samples collected prior to treatment. We observed a two- to sixfold increased platelet desialylation in patients with anti-GPIbα antibodies (*n* = 9) compared to that in patients with anti-GPIIbIIIa (*n* = 14) or without detectable antibodies (*n* = 10) (Table 1 and Fig. 1B). However, statistical significance was not reached, which is likely due to small sample size. Future larger studies should be useful in determining direct correlation between anti-GPIbα antibody positivity with platelet desialylation.

Interestingly, we also observed significant platelet desialylation in patients with non-ITP thrombocytopenias including connective tissue diseases (CTD), myelodysplastic syndrome (MDS), and aplastic anemia (AA) (*p* < 0.001) but not acute myeloid leukemia (AML) compared to healthy controls (Table 1 and Fig. 1B). Notably, although RCA-1 and ECL measures different types of deglycosylation, we did not observe significant difference between these two assays, suggesting either of them can be used for the potential diagnosis and prognosis.

In summary, our data demonstrates for the first time that the higher level of platelet desialylation is correlated with non-response to the first-line ITP therapies (likely also splenectomies; Additional file 1: Supplementary Material). These findings not only suggest that platelet desialylation is a useful biomarker in predicting response to treatment in clinical ITP but positions sialidase inhibitors, such as Tamiflu [12], as a potential novel therapeutic in the treatment of ITP as well as other thrombocytopenias.

## Additional file

Additional file 1: Supplementary Material. (DOCX 49 kb)

## Abbreviations

AA: Aplastic anemia
AML: Acute myeloid leukemia
CR: Complete responder
CTD: Connective tissue disease
ECL: *Erythrina cristagalli* lectin
FITC: Fluorescein isothiocyanate
ITP: Immune thrombocytopenia
IVIG: Intravenous immunoglobulin G
MAIPA: Monoclonal antibody immobilization of platelet antigen assay
MDS: Myelodysplastic syndrome
NR: Non-responder
PRP: Platelet-rich plasma
R: Responder
RCA-1: *Ricinus communis* agglutinin I
