# Supplementary material for: Platelet desialylation correlates with efficacy of first-line therapies for immune thrombocytopenia
Source: J Hematol Oncol. 2017 Feb 8;10:46. doi: 10.1186/s13045-017-0413-3 (PMC5304552; doi:10.1186/s13045-017-0413-3)
Supplement: Supplementary file 1 — Supplementary Material. (DOCX 49 kb) [file 13045_2017_413_MOESM1_ESM.docx]

***Supplementary Material***

**Materials and Methods**

**Study Design and Patients**

ITP and non-ITP patients, and healthy volunteers were enrolled at the Second Affiliated Hospital of Anhui Medical University, Hefei, China. All participants signed a statement of written informed consent. The procedures described in this study were all approved by the ethics committee of Anhui Medical University. Sixty-one diagnosed primary ITP patients (**Table 1**) who were treated between 2013 and 2015 at the Hospital were randomly and consecutively enrolled. ITP patients with secondary thrombocytopenia as induced by other hematological diseases, infections, endocrine system diseases, cancer, etc. were excluded. Concurrently, secondary ITP patients and non-immune thrombocytopenic patients including connective tissue disease (CTD), myelodysplastic syndrome (MDS), aplastic anemia (AA) and acute myeloid leukemia (AML) patients were also enrolled. Twenty healthy human volunteers were enrolled as control (**Table 1**).

Improved monoclonal antibody immobilization of platelet antigen assay (MAIPA) [1, 2] was used to detect anti-GPIIbIIIa and/or GPIbα specific autoantibodies. Due to the specimen retention difficulties, only 33 patients’ blood samples were available for MAIPA. These 33 samples were divided into three groups: 1) anti-GPIbα antibody positive (+) group, which include both single anti-GPIbα antibody positive samples and the samples positive for both anti-GPIbα and anti-GPIIbIIIa (our earlier studies reported that anti-GPIbα, but not anti-GPIIbIIIa, dictates the therapeutic efficacy when patients contain both anti-GPIbα and anti-GPIIbIIIa antibodies)[1, 3]; 2) single anti-GPIIb/IIIa antibody (+) group, which are positive for anti-GPIIbIIIa without detectable anti-GPIbα antibodies; and 3) the double negative group, which do not contain detectable antibodies to either GPIbα or GPIIbIIIa.

**Treatment**

All enrolled primary ITP patients underwent a first-line treatment regimen of high-dose dexamethasone (40mg/d, 3 - 5 days) with/without high-dose IVIG (0.4g/kg/day, 3 - 5 days) followed by daily low-dose prednisone (1mg/kg) with subsequent dose adjustments based on efficacy. Treatments continued for one month with regular blood cell counts. Two patients underwent splenectomy, and 1 patient had splenic embolization (**Supplementary Table 1**). The efficacy of treatment was evaluated based on standards of international working group as: 1) Complete response (CR): platelet count ≥100×10^9^/L and without bleeding. 2) Response (R): platelet count ≥30×10^9^/L and at least 2-fold increase from baseline count and no bleeding. 3) No response (NR): Platelet count <30×10^9^/L or less than 2-fold increase from baseline or bleeding [4].

**Blood collection and platelet isolation**

Venous blood was obtained from study participants via venipuncture into 3.2% trisodium citrate [5-7]. Platelet-rich plasma (PRP) was obtained by centrifugation for 15 min at 160g, no break, 22℃ [1, 8, 9]. Washed-platelets from PRP was obtained by centrifugation at 340 g for 15 mins, 22℃ with 10 ng/mL PGI_2_ and re-suspended in HEPES Tyrode’s buffer (140 mM NaCl， 0.5 mM NaHCO_3_，3 mM KCl，0.5 mM MgCl_2_，10 mM HEPES，10 mM glucose，PH =7.4). Platelets were counted and adjusted to a final concentration of 200 × 10^6^ /mL，and left at 22°C for 30 minutes before experiments

**Flow cytometry**

Platelet desialylation was assessed by Fluorescein isothiocyanate (FITC) conjugated Ricinus communis agglutinin I (RCA-1) and FITC-conjugated Erythrina cristagalli lectin (ECL) which binds to exposed surface galactose and β-GlcNAc residues, respectively [10-12]. RCA-1 and ECL (0.2ug/mL) were incubated with platelets for 20 mins at 22℃ and detected with flow cytometry [13, 14]. Overall desialylation level of platelets is represented as percentage of RCA-1and ECL positive platelets among whole platelet population.

**Statistical analysis**

SPSS17.0 software was used for statistical analysis. Normal distribution measurement data is presented as mean ± SEM, skewed distribution measurement data is presented as M (P25, P75), in which M represents the median, P25 and P75 represent the 25th percentile and 75th percentile respectively. Statistically significant differences among groups were assessed by non-parametric test (Kruskal-Wallis rank sum test). Correlation between platelet desialylation and response to the therapies was analyzed by Spearman’s rank correlation coefficient. A *p* value of less than 0.05 was considered significant. Statistical analyses were performed using Prism software (GraphPad).

**Table S1. Clinical data of the three surgery patients**

| Surgical approach | Age | Gender | Platelet number  (×10^9^ /L) | Antibody | RCA-1(%) | ECL(%) | Platelet number after the therapy  (×10^9^ /L) |
| --- | --- | --- | --- | --- | --- | --- | --- |
| Splenic embolization | 41 | Female | 18 | Anti-GPIIbIIIa(+) | 5.7 | 31.9 | 7 |
| splenectomy | 45 | Female | 6 | Anti-GPIbα(+) | 44.4 | 20.4 | 15 |
| splenectomy | 34 | Female | 3 | Anti-GPIbα(+) | 2.5 | 0.6 | 233 |

**Supplementary Table 1 Legend**

Three patients of the current study, who did not respond to glucocorticoid and IVIG therapy, received splenectomy or splenic embolization. One of them had no significant higher platelet desialylation. After splenectomy, she achieved complete response, suggesting her platelets were cleared mainly in the spleen. Another two patients had higher platelet desialylation. Unfortunately, no significant platelet count increased after their surgeries, suggesting that their platelet clearance is not via the conventional Fc receptor-dependent pathway in spleens, and the hepatic clearance pathway may predominate.

**References for supplementary data*:***

1. Zeng Q, Zhu L, Tao L, et al. Relative efficacy of steroid therapy in immune thrombocytopenia mediated by anti-platelet GPIIbIIIa versus GPIbalpha antibodies. Am J Hematol 2012;87:206-208.

2. Brighton TA, Evans S, Castaldi PA, et al. Prospective evaluation of the clinical usefulness of an antigen-specific assay (MAIPA) in idiopathic thrombocytopenic purpura and other immune thrombocytopenias. Blood 1996;88:194-201.

3. Peng J, Ma SH, Liu J, et al. Association of autoantibody specificity and response to intravenous immunoglobulin G therapy in immune thrombocytopenia: a multicenter cohort study. Journal of thrombosis and haemostasis : JTH 2014;12:497-504.

4. Rodeghiero F, Stasi R, Gernsheimer T, et al. Standardization of terminology, definitions and outcome criteria in immune thrombocytopenic purpura of adults and children: report from an international working group. Blood 2009;113:2386-2393.

5. Jansen AJ, Josefsson EC, Rumjantseva V, et al. Desialylation accelerates platelet clearance after refrigeration and initiates GPIbalpha metalloproteinase-mediated cleavage in mice. Blood 2012;119:1263-1273.

6. Hoffmeister KM, Josefsson EC, Isaac NA, et al. Glycosylation restores survival of chilled blood platelets. Science 2003;301:1531-1534.

7. Xu X, Wu J, Zhai Z, et al. A novel fibrinogen Bbeta chain frameshift mutation in a patient with severe congenital hypofibrinogenaemia. Thrombosis and haemostasis 2006;95:931-935.

8. Wang Y, Reheman A, Spring CM, et al. Plasma fibronectin supports hemostasis and regulates thrombosis. The Journal of clinical investigation 2014;124:4281-4293.

9. Wang Y, Vachon E, Zhang J, et al. Tyrosine phosphatase MEG2 modulates murine development and platelet and lymphocyte activation through secretory vesicle function. The Journal of experimental medicine 2005;202:1587-1597.

10. Alioglu B, Tasar A, Ozen C, et al. An experience of oseltamivir phosphate (tamiflu) in a pediatric patient with chronic idiopathic thrombocytopenic purpura: a case report. Pathophysiol Haemost Thromb 2010;37:55-58.

11. Jansen AJ, Peng J, Zhao HG, et al. Sialidase inhibition to increase platelet counts: A new treatment option for thrombocytopenia. Am J Hematol 2015;90:E94-95.

12. Li J, van der Wal DE, Zhu G, et al. Desialylation is a mechanism of Fc-independent platelet clearance and a therapeutic target in immune thrombocytopenia. Nat Commun 2015;6:7737.

13. Yang H, Lang S, Zhai Z, et al. Fibrinogen is required for maintenance of platelet intracellular and cell-surface P-selectin expression. Blood 2009;114:425-436.

14. Zhai Z, Wu J, Xu X, et al. Fibrinogen controls human platelet fibronectin internalization and cell-surface retention. Journal of thrombosis and haemostasis : JTH 2007;5:1740-1746.
